# Low-dose Interleukin-2 For Psoriasis Therapy Based on the Regulation of Th17/Treg Cell Balance in Peripheral Blood

**DOI:** 10.1007/s10753-023-01883-6

**Published:** 2023-08-18

**Authors:** Zusha Qiao, Wenpeng Zhao, Yan Liu, Wenli Feng, Yan Ma, Hongzhong Jin

**Affiliations:** 1 Department of Dermatology, Second Hospital of Shanxi Medical University, Taiyuan, China; 2 Department of Dermatology, State Key Laboratory of Complex Severe and Rare Diseases, Peking Union Medical College Hospital, Chinese Academy of Medical Sciences and Peking Union Medical College, National Clinical Research Center for Dermatologic and Immunologic Diseases, Beijing, 100730 China; 3 Department of Rheumatology, Second Hospital of Shanxi Medical University, Taiyuan, China; 4 Department of Cancer prevention and control office, Shanxi Province Cancer Hospital/Shanxi Hospital Affiliated to Cancer Hospital, Chinese Academy of Medical Sciences/Cancer Hospital Affiliated to Shanxi Medical University, Taiyuan, China

**Keywords:** interleukin-2, immunotherapy, treg cells, psoriasis

## Abstract

The imbalance between regulatory T (Treg) cells and efficient T cells plays an important role in psoriasis. Low-dose interleukin (IL)-2 can preferentially activate Treg cells and ameliorate the imbalance of Treg/efficient T cells. This study focused on the status of circulating CD4^+^ T subsets and the clinical efficacy of low-dose IL-2 therapies in psoriasis. This retrospective study included peripheral blood samples obtained from 45 psoriatic patients and 40 healthy controls. The 45 psoriatic patients received three cycles of subcutaneous low-dose IL-2 treatment (0.5 million IU/day for 2 weeks) combined with conventional therapies. Inflammatory indices, CD4^+^ T-lymphocyte subsets, and cytokines were measured in all patients before and after treatment. The percentage of Treg cells was dramatically decreased in the psoriasis group compared to the healthy group, and the percentage of Treg cells negatively correlated with the disease indices and the Psoriasis Area and Severity Index (PASI) (*P* < 0.001). The Th17/Treg ratio was significantly increased in the psoriasis group compared to the healthy group, and the Th17/Treg ratio positively correlated with disease indices and PASI (*P* < 0.001). Low-dose IL-2 treatment significantly amplified the percentage of Treg cells and restored the Th17 and Treg immune balance in psoriasis (*P* < 0.001). Low-dose IL-2 combination therapy effectively improved the clinical manifestations of psoriasis but decreased the inflammatory indicators of the disease activity, with no apparent side effects. Thus, low-dose IL-2 provides a new strategy for the treatment of psoriasis.

**Supplementary Information:**

The online version contains supplementary material available at 10.1007/s10753-023-01883-6.

## INTRODUCTION

Psoriasis is an immune-mediated systemic inflammatory disease. Its aetiology and pathogenesis are not fully understood. The imbalance between T-helper 1 (Th1) and T-helper 2 (Th2) cells is considered as the main pathogenesis of psoriasis, and growing evidence suggests that T-helper 17 (Th17) cells and CD4^+^CD25^+^ regulatory T (Treg) cells play important roles in the pathogenesis of psoriasis [1–4].

Interleukin-2 (IL-2) is an important immune regulatory factor. High-dose IL-2 has anti-tumour and enhanced immune effects, while low-dose IL-2 has immunosuppressive effects. Treg cells express CD25 on their surfaces and can bind the IL-2 receptor (α, β, and γ) with high affinity. Low-dose IL-2 can activate Treg cells, while other subsets of T cells require higher doses of IL-2 for activation. This forms the molecular basis of the low-dose IL-2 regulation of Treg cell functions [5]. Although the role of low-dose IL-2 in the treatment of autoimmune diseases has also attracted more attention, there are rare direct studies on the efficacy of low-dose IL-2 in psoriasis.

In this study, we evaluated the Th1, Th2, Th17, and Treg cells, cytokines, and inflammatory indices in patients with different types of psoriasis to explore the roles of Th1/Th2 and Th17/Treg imbalance in the pathogenesis of psoriasis. To investigate new concepts for the treatment of psoriasis, we observed the inflammatory and immunological indices of patients with psoriasis before and after treatment with low-dose IL-2 to clarify the clinical efficacy and safety.

## METHODS

### Subjects

In this retrospective study, peripheral blood samples were obtained from 45 psoriatic patients (24 men and 21 women; 21–58 years of age; 17 psoriasis vulgaris (PV), 18 psoriasis arthritis (PA), 5 erythrodermic psoriasis (EP), and 5 generalized pustular psoriasis (GPP)) and 40 healthy controls (19 men and 21 women; 23–53 years of age) from February 2020 to November 2021. The inclusion criteria were a documented diagnosis of psoriasis and a Psoriasis Area and Severity Index (PASI) ≥ 10. The exclusion criteria were another severe or progressive autoimmune/inflammatory disease; haematological disorders; vital organ failure; cancer; and active HIV, hepatitis B virus, or Epstein–Barr virus infections.

### Study Design and Treatment

The 45 psoriatic patients received methotrexate at 7.5 mg per week for 12 weeks to control disease activity. From 13 to 24 weeks, the patients received conventional therapy combined with three cycles of low-dose IL-2 therapy using recombinant human IL-2 (rhIL-2, Beijing, China). Each of the three cycles consisted of daily subcutaneous injections of IL-2 for 2 consecutive weeks, followed by a 2-week break. The daily dose in every cycle was 0.5 million international units (MIU) of IL-2 for all patients. This study was conducted in accordance with the *Declaration of Helsinki* and approved by the Ethics Committee of the Second Hospital of Shanxi Medical University (No. 2021-YX-010). Written informed consent was obtained from all participates prior to their enrolment in this study.

### Measurement of Inflammatory Indices

The peripheral neutrophil count, leukocyte count, lymphocyte count, platelet count, neutrophil-to-lymphocyte ratio (NLR), platelet-to-lymphocyte ratio (PLR), erythrocyte sedimentation rate (ESR), and c-reactive protein (CRP) concentration in *serum* were recorded.

### Blood Sample Collection and Preparation

Peripheral blood mononuclear cells (PBMCs) were isolated from heparinized peripheral blood by density gradient centrifugation with Ficoll–Paque PREMIUM 1.077 (GE Healthcare Life Sciences, Pittsburgh, PA). The cells were then resuspended in RPMI 1640 medium containing phorbol 12-myristate13-acetate (PMA) (50 ng/ml), GolgiStop (1 µl), and ionomycin (1 µg/ml) for 5 h in an incubator at 37 °C with 5% CO_2_.

### Cell Staining

The stimulated cells were stained with fluorescein isothiocyanate (FITC)–labelled CD4 antibody for 30 min at 4 °C. After surface staining, the cells were fixed, permeabilized, and stained with interferon-γ (IFN-γ)-phycoerythrin (PE) (Th1), IL-4-PE (Th2), and IL-17-PE (Th17). For Treg cells, the PBMCs were incubated with FITC–labelled CD4 and allophycocyanin (APC)-labelled CD25 antibodies at 4 °C for 30 min. The cells were then treated with 1 ml of freshly prepared fixation/permeabilization solution followed by staining with PE-labelled Foxp3 antibody.

### Flow Cytometric Analysis

The percentages of Th1, Th2, Th17, and Treg cells were detected by flow cytometry (Calibur, BD, USA) and analysed by fluorescence-activated cell sorting (FACS) (Canto II, BD Biosciences, San Jose, CA) followed by data analysis using FlowJo 7.6 software (TreeStar, San Carlos, CA).

### Cytometric Bead Array

The cytokines in* serum* from peripheral blood were analysed using a cytometric bead array human Th1, Th2, Th17, and Treg cytokine kit (Becton Dickinson, USA) along with a FACS Calibur flow cytometer. The beads were coated with antibodies that specifically reacted with each of the cytokines to be detected in the multiplex system. The beads could be differentiated by their sizes and their distinct spectral addresses. The *serum* concentrations of IL-2, IL-4, IL-6, IL-10, IL-17, IFN-γ, and tumour necrosis factor (TNF)-α were determined using the cytometric bead array technique.

### Evaluation of Therapeutic Efficiency

Therapeutic efficiency was primarily evaluated using the quantitative PASI score, which measures the severity of psoriatic lesions based on area coverage and plaque appearance, including scaling, infiltration, and erythema. PASI 50 and PASI 75 indicated 50% and 75% improvements in PASI, respectively. PA patients were identified according to the criteria of the American College of Rheumatology (ACR20 response). At weeks 0 and 24 of treatment, all observation indices were reviewed, and the adverse reactions of patients were recorded in detail.

### Statistical Analysis

SPSS version 22.0 was used for all data analyses (SPSS Inc., Chicago, IL, USA), and *P* < 0.05 was considered statistically significant. Continuous variables were expressed as mean ± standard deviation, and the two groups were compared using Student’s *t*-test. One-way analysis of variance and least significant difference (LSD) test were used to analyse multiple groups. Median (interquartile range) and Wilcoxon test were used for abnormally distributed data when there were two groups. Kruskal–Wallis and LSD tests were used for multiple groups. For categorical variables, counts (percentages) were described and compared using the *χ*^2^ test. Relationships between two variables were evaluated by Spearman correlation analysis.

## RESULTS

### Baseline Characteristics of All Subjects

In total, 45 psoriatic patients and a group of 40 healthy subjects were enrolled. The difference between the ages of the psoriasis and healthy groups was not statistically significant (37.67 ± 5.54 vs. 36.00 ± 8.66, respectively; *P* > 0.05). The percentage of men in the psoriasis group was not significantly different from that in the healthy group (52.12% vs. 47.5%, respectively; *P* > 0.05). The years of median disease duration were 6.0 (7.5) in the psoriasis group (*P* < 0.001). The median PASI score was 19.500 (11.900) in the psoriasis group (*P* < 0.001; Table 1).

**Table 1:** Baseline Characteristics and Inflammatory Parameters of All Subjects [mean ± SD, M(IQR)]

**Parameter**	**Healthy groups**	**Psoriasis groups**	***t/χ***^***2***^	***P***
N	40	45	_**-**_	_**-**_
Age (year)	36.000 ± 8.659	37.667 ± 9.535	–0.840	0.403
Men (%)	47.500	53.300	0.288	0.591
Duration (year)	0.000 (0.000)	6.000 (7.500)	8.387	< 0.001
PASI	0.000 (0.000)	19.500 (11.900)	–13.871	< 0.001
Leukocytes (× 10^9^/L)	6.798 ± 2.064	7.030 (3.410)	1.985	0.047
Neutrophil (× 10^9^/L)	4.566 ± 1.951	4.920 (3.140)	2.157	0.031
Lymphocytes (× 10^9^/L)	1.946 ± 0.520	1.947 ± 0.540	–0.007	0.994
Platelets (× 10^9^/L)	235 ± 60	235 (189)	1.444	0.149
ESR (mm/h)	11 (6)	30 (63)	4.949	< 0.001
CRP (mg/L)	5.740 (5.530)	18.560 (43.645)	5.212	< 0.001
NLR	2.105 (1.416)	3.440 (1.670)	5.115	< 0.001
PLR	127.941 ± 43.173	144.630 (96.175)	1.259	0.208

*NLR* neutrophil-to-lymphocyte ratio, *PASI* Psoriasis Area and Severity Index, *PLR* platelet-to-lymphocyte ratio

The psoriasis group included 17 PV, 18 PA, 5 EP, and 5 GPP patients. The ages and sexes of the four types in the psoriasis group were not significant (*P* > 0.05). The PASI scores of the different psoriasis groups are shown in Table 2 (*P* < 0.001; Table 2).

**Table 2:** Clinical Characteristics and Inflammatory Parameters Among the Different Psoriasis Groups [Mean ± SD, M(IQR)]

**Parameter**	**Healthy groups**	**PV**	**PA**	**EP**	**GPP**	***F/H***	***P***
N	40	17	18	5	5	_-_	_-_
Age (year)	36.000 ± 8.659	37.059 ± 9.202	35.278 ± 9.809	42.200 ± 8.408	43.800 ± 9.039	1.406	0.240
Men (%)	47.500	52.941	55.556	60.000	40.000	0.773	0.942
Duration (year)	0.000 (0.000)^*^	6.147 ± 0.803^*^	5.000 (8.500)^*^	11.200 ± 1.985^*^	7.000 ± 2.168^*^	71.581	< 0.001
PASI	0.000 (0.000)^*^	18.171 ± 1.490^*^	14.250 (9.550)^*^	42.700 ± 2.337^*^	20.620 ± 3.896^*^	74.417	< 0.001
Leukocytes (× 10^9^/L)	6.798 ± 2.064^*^	6.871 ± 1.671^*^	6.806 ± 1.435^*^	17.792 ± 4.086^*^	10.536 ± 1.926^*^	37.147	< 0.001
Neutrophil (× 10^9^/L)	4.566 ± 1.951^*^	4.634 ± 1.397^*^	4.679 ± 1.451^*^	14.874 ± 3.985^*^	8.398 ± 1.551^*^	37.473	< 0.001
Lymphocytes (× 10^9^/L)	1.946 ± 0.520	1.971 ± 0.704	1.965 (0.350)	2.004 ± 0.615	1.988 ± 0.518	0.212	0.995
Platelets (× 10^9^/L)	235 ± 60^*^	239 (64)^*^	263 ± 101^*^	460 ± 100^*^	316 ± 83	15.410	0.004
ESR (mm/h)	12 (6)^*^	19 ± 11^*^	44 ± 34^*^	118 ± 48^*^	82 ± 23^*^	38.109	< 0.001
CRP (mg/L)	5.740 (5.530)^*^	8.330 (11.135)^*^	16.945 (40.160)^*^	73.484 ± 9.991^*^	65.334 ± 58.328^*^	42.070	< 0.001
NLR	2.105 (1.416)^*^	3.306 ± 0.815^*^	3.292 ± 0.734^*^	9.180 ± 2.262^*^	5.196 ± 0.925^*^	36.933	< 0.001
PLR	127.941 ± 43.173^*^	139.940 (70.320)^*^	146.414 ± 75.668^*^	240.004 ± 62.587^*^	162.852 ± 41.455	12.680	0.013

*NLR* neutrophil-to-lymphocyte ratio, *PASI* Psoriasis Area and Severity Index, *PLR* platelet-to-lymphocyte ratio

*P < 0.05

### NLR and PLR Combined With ESR and CRP May Indicate Systemic Inflammation and Immune Status in Psoriasis

Compared with the healthy group, circulating leukocyte count, neutrophil count, NLR, ESR, and CRP were higher in the psoriasis group (*P* < 0.05). No significant differences were observed in lymphocyte count, platelet count, and PLR between the psoriasis and healthy groups (*P* > 0.05; Table 1). In the four types of the psoriasis group, PLR, platelet count, leukocyte count, and neutrophil count in peripheral blood were significantly increased in EP patients compared to PV patients, PA patients, and the healthy group (*P* < 0.05). The ESR and the CRP *serum* concentration were significantly higher in PA, EP, and GPP patients than in the healthy group (*P* < 0.001). No significant differences were observed in lymphocyte count among the psoriasis groups (*P* > 0.05; Table 2).

### Differences in CD4^+^ T Cell Subset Numbers and Cytokines Between the Psoriasis and Healthy Groups

#### The Psoriasis Group Was Associated With a Higher Proportion of Th17 Cells, a Lower Proportion of Treg Cells, and an Increased Th17/Treg Ratio Compared to the Healthy Group

**Fig. 1:**
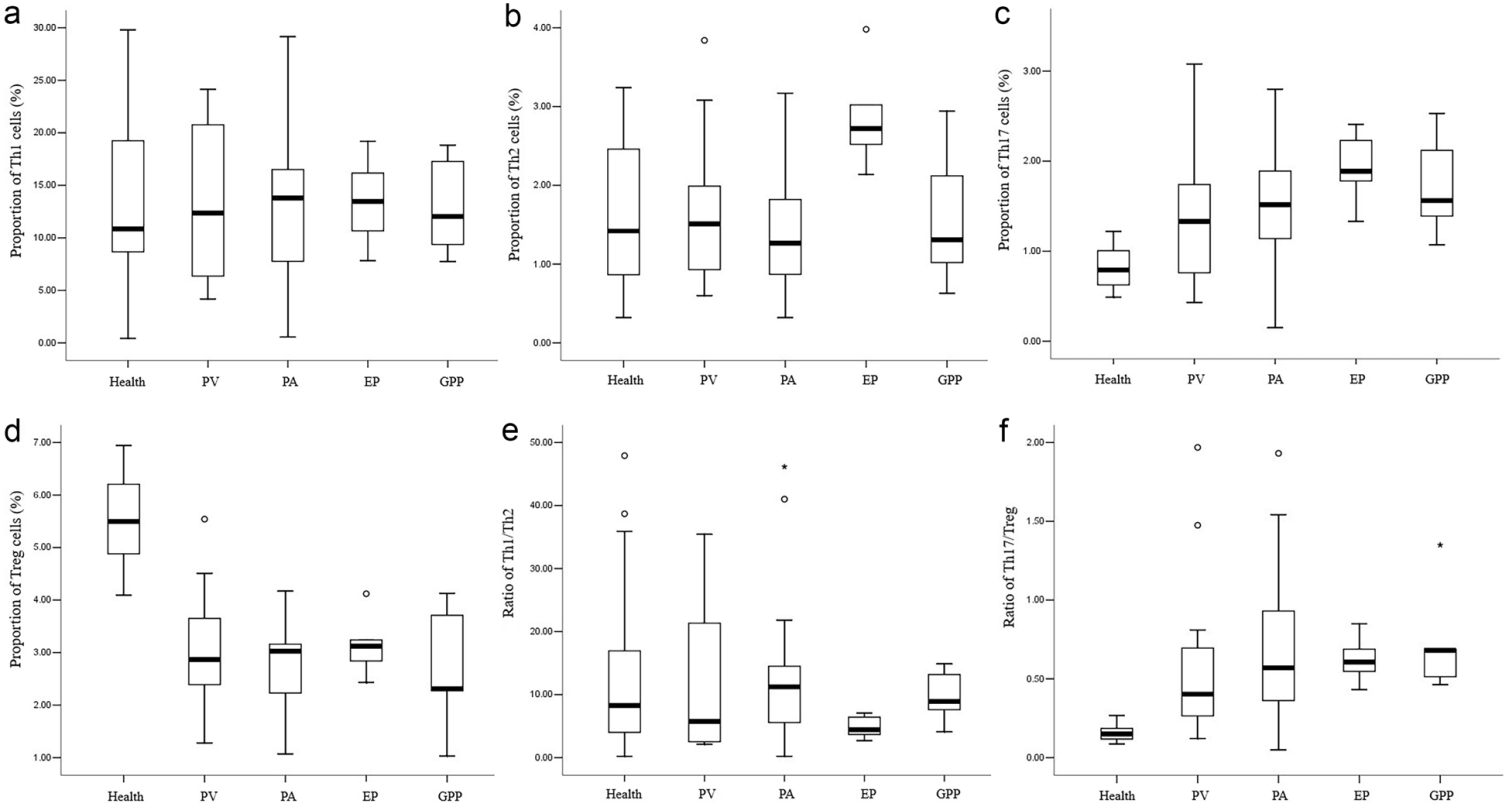
**a** Proportion of Th1 cells. **b** Proportion of Th2 cells. **c** Proportion of Th17 cells. **d** Proportion of Treg cells. **e** Ratio of Th1/Th2. **f** Ratio of Th17/Treg.

The proportion of Th17 cells in peripheral blood was significantly higher in the psoriasis group compared with the healthy group (*P* < 0.001; Table 3), which increased in the following order: PV < PA < GPP < EP (*P* < 0.001; Fig. 1c). In contrast, the percentage of Treg cells was dramatically lower in the psoriasis group compared to the healthy group (*P* < 0.001; Table 3), which increased in the following order: GPP < PA < PV < EP (*P* < 0.001; Fig. 1d). As a result, the Th17/Treg ratio was increased in the psoriasis group compared with the healthy group (*P* < 0.001; Table 3), which increased in the following order: PV < PA < EP < GPP (*P* < 0.001; Fig. 1f). However, no significant differences in Th1, Th2, or the Th1/Th2 ratio were observed between the psoriasis and healthy groups (*P* > 0.05, Table 3; Fig. 1a, b and e).

**Table 3:** Differences in CD4^+^ T Cell Subset Numbers in the Psoriasis and Healthy Group [Mean ± SD, M(IQR)]

**Group**	**Healthy group**	**Psoriasis group**	***t/Z***	***P***
N	40	45	-	-
Th1 (%)	13.235 ± 6.835	13.114 ± 6.864	0.081	0.935
Th2 (%)	1.420 (1.618)	1.510 (1.405)	−0.445	0.657
Th17 (%)	0.826 ± 0.215	1.546 ± 0.681	−6.728	< 0.001
Treg (%)	5.483 ± 0.802	2.859 ± 0.991	13.314	< 0.001
Th1/Th2	8.273 (13.898)	7.598 (11.016)	71.581	74.417
Th17/Treg	0.154 ± 0.045	0.547 (0.364)	−6.947	< 0.001

#### Psoriasis Decreased IL-10 Concentrations, Increased IL-17 Concentrations, and Increased the IL-17/IL-10 Ratio Compared to the Healthy Group

**Fig. 2:**
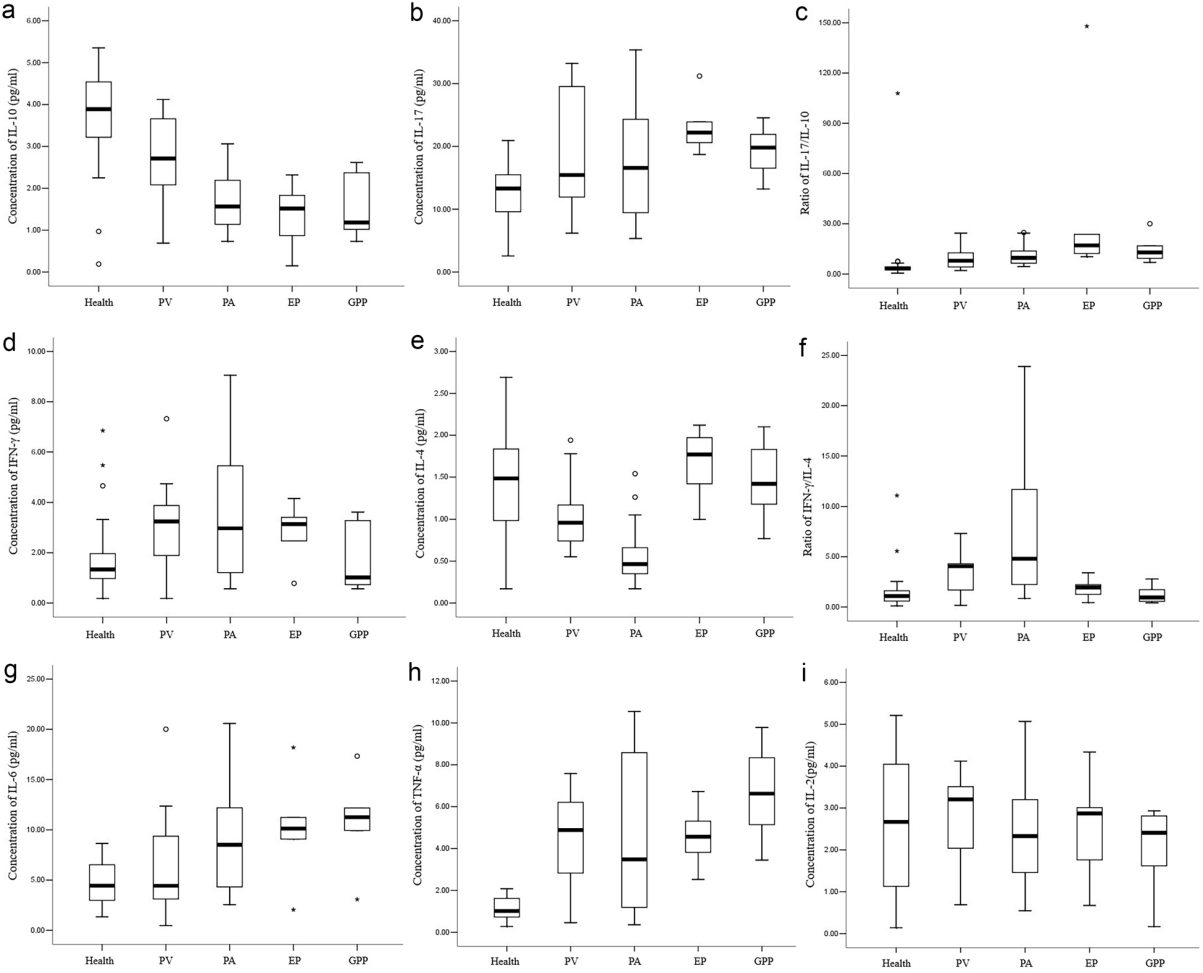
Concentrations of **a** IL-10, **b** IL-17, **d** IFN-γ, **e** IL-4, **g** IL-6, **h** TNF-α, and **i** IL-2. Ratios of **c** IL-17/IL-10 and **f** IFN-γ/IL-4.

The *serum* concentrations of IL-10 in peripheral blood were lower in the psoriasis group than in the healthy group (*P* < 0.001; Table 4), which decreased in the following order: GPP < EP < PA < PV (*P* < 0.001; Fig. 2a). In contrast, the *serum* concentrations of IL-17 were higher in the psoriasis group compared to the healthy group (*P* < 0.001; Table 4), which decreased in the following order: PV < PA < EP, whereas the difference in IL-17 between the GPP patients and the healthy group was not significant (*P* > 0.05; Fig. 2b). It may be related to the difference in the distribution of IL-17 in peripheral blood and local tissues and the small sample size of GPP patients. As a result, the IL-17/IL-10 ratio was increased in the psoriatic patients compared with the healthy group (*P* < 0.001; Table 4), which increased in the following order: PV < PA < GPP < EP (*P* < 0.001; Fig. 2c).

**Table 4:** Differences in Cytokine Levels Between the Psoriasis and Healthy Groups (pg/ml) [Mean ± SD, M(IQR)]

**Group**	**Healthy group**	**Psoriasis group**	***t/Z***	***P***
N	40	45	-	-
IL-10	3.807 ± 1.094	2.012 ± 0.973	8.005	< 0.001
IL-17	12.778 ± 4.395	19.262 ± 8.678	–4.415	< 0.001
IFN-γ	1.335 (1.040)	3.007 ± 2.069	3.002	0.003
IL-4	1.454 ± 0.565	0.976 ± 0.551	3.948	< 0.001
IL-6	4.753 ± 2.207	8.330 (7.500)	3.372	0.001
TNF-α	1.168 ± 0.521	4.888 ± 2.824	–8.673	< 0.001
IL-2	2.670 (2.953)	2.518 ± 1.219	–0.735	0.462
IL-17/IL-10	3.247 (2.027)	10.644 (8.780)	6.084	< 0.001
IFN-γ/IL-4	1.097 (1.025)	3.019 (4.321)	4.393	< 0.001

#### Psoriasis Increased IFN-γ Concentrations, Decreased IL-4 Concentrations, and Increased the IFN-γ/IL-4 Ratio Compared to the Healthy Group

Compared with the healthy group, the IFN-γ concentrations were significantly elevated in the psoriasis group (*P* = 0.003). In contrast, the *serum* concentrations of IL-4 were significantly lower in the psoriasis group than in the healthy group (*P* < 0.001). As a result, the IFN-γ/IL-4 ratio was higher in the psoriasis group than in the healthy group (*P* < 0.001; Table 4). PV and PA patients had increased IFN-γ concentrations, decreased IL-4 concentrations, and greatly increased IFN-γ/IL-4 ratio compared to the healthy group. In contrast, no significant differences in IFN-γ, IL-4, or the IFN-γ/IL-4 ratio were observed between the EP and GPP patients compared with the healthy group (*P* > 0.05; Fig. 2d–f).

#### IL-6 and TNF-α Concentrations Were Significantly Elevated in Psoriatic Patients Compared to the Healthy Group

IL-6 is a critical factor in the homeostatic balance between Th17 and Treg cells. The *serum* concentrations of IL-6 were significantly elevated in PA, EP, and GPP patients compared with the healthy group, which was the highest in GPP patients (*P* < 0.001). However, the difference in IL-6 between the PV patients and the healthy group was not significant (*P* > 0.05; Fig. 2g).

The TNF-α concentrations were significantly increased in the psoriatic patients compared with the healthy group (*P* < 0.001; Table 4), which increased in the following order: PA < EP < PV < GPP (*P* < 0.001; Fig. 2h). The IL-2 levels in the psoriasis group were not significantly different than those in the healthy group (*P* > 0.05, Table 4; Fig. 2i).

### Correlation Analysis of CD4^+^ T Lymphocyte Subsets With Inflammatory Indicators in Psoriasis

**Fig. 3:**
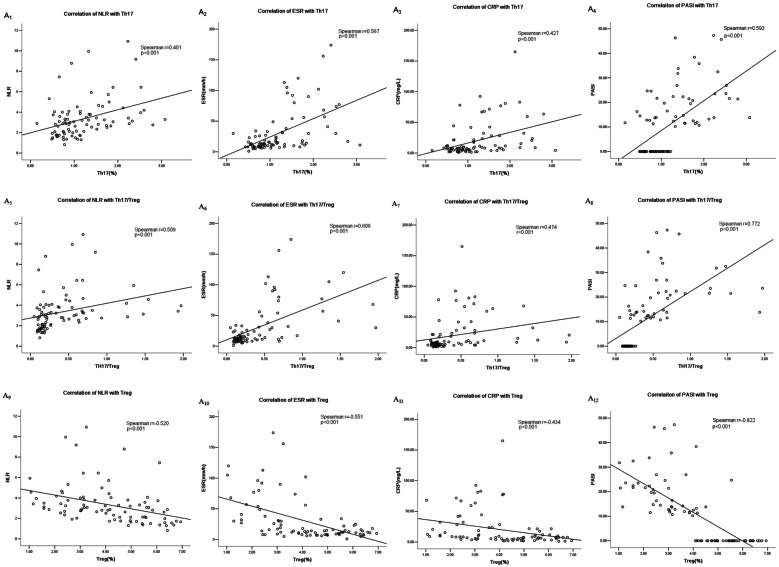

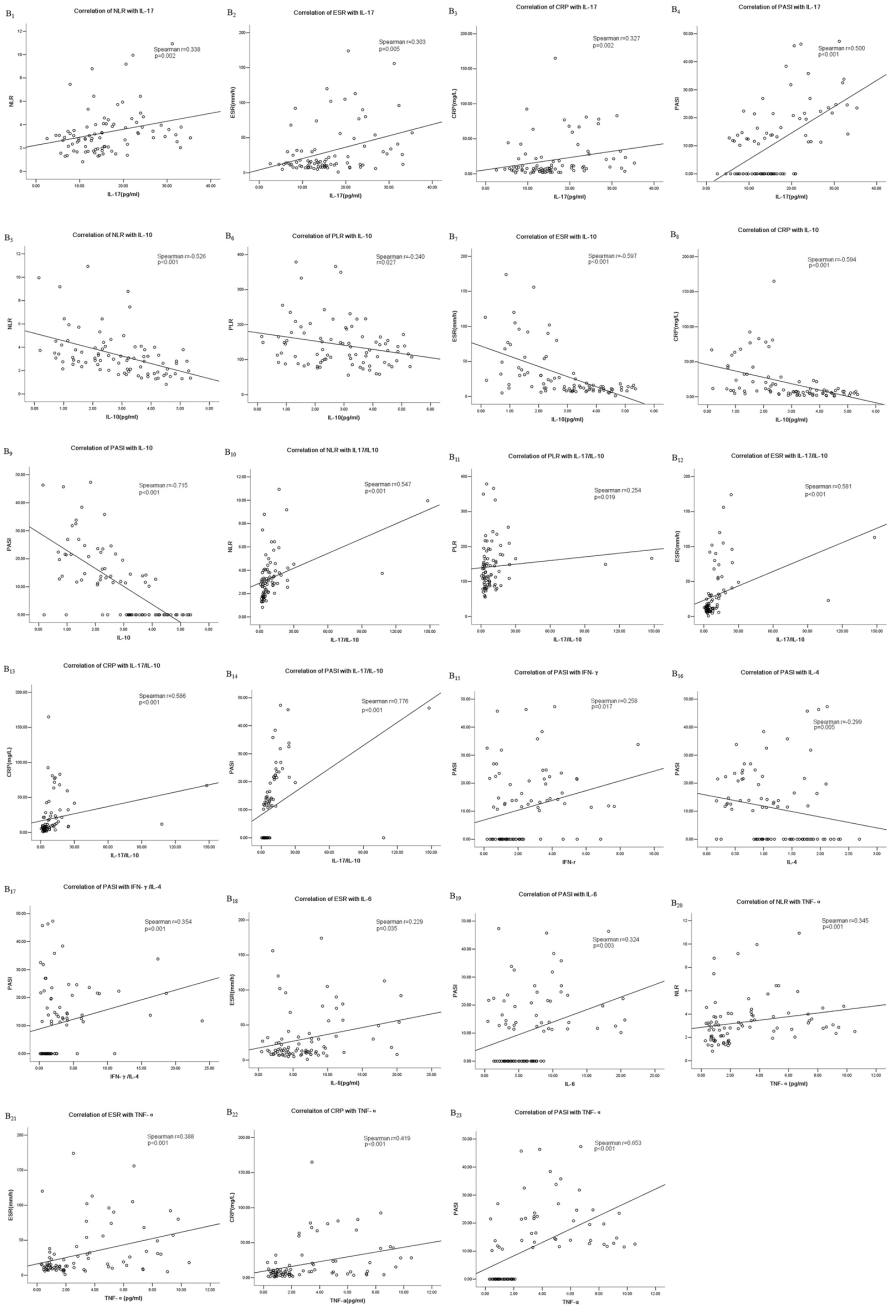
**A1**–**A12** Correlation analysis of CD4^+^ T lymphocyte subsets with inflammatory indicators in psoriasis. **B1**–**B23** Correlation analysis of cytokines with inflammatory indicators in psoriasis.

The percentage of Th17 cells positively correlated with NLR, ESR, CRP, and PASI (*P* < 0.001; Fig. 3A1–A4). The percentage of Treg cells negatively correlated with NLR, ESR, CRP, and PASI (*P* < 0.001; Fig. 3A9–A12). The Th17/Treg ratio positively correlated with NLR, ESR, CRP, and PASI (*P* < 0.001; Fig. 3A5–A8). The inflammatory indices did not significantly correlate with the Th1 percentage, Th2 percentage, or Th1/Th2 ratio (*P* > 0.05).

### Correlation Analysis of Cytokines With Inflammatory Indicators in Psoriasis

The IL-17 *serum* concentrations positively correlated with NLR, ESR, CRP, and PASI (*P* < 0.001; Fig. 3B1–B4). The *serum* concentrations of IL-10 negatively correlated with NLR, PLR, ESR, CRP, and PASI (*P* < 0.001; Fig. 3B5–B9). The IL-17/IL-10 ratio positively correlated with NLR, PLR, ESR, CRP, and PASI (*P* < 0.001; Fig. 3B10–B14).

The *serum* concentrations of IFN-γ and the IFN-γ/IL-4 ratio positively correlated with PASI (*P* < 0.001; Fig. 3B15, B17). The *serum* concentrations of IL-4 negatively correlated with PASI (*P* < 0.001; Fig. 3B16). The *serum* concentrations of IL-6 positively correlated with ESR and PASI (*P* < 0.001; Fig. 3B18, B19). The TNF-α *serum* concentrations positively correlated with NLR, ESR, CRP, and PASI (*P* < 0.001; Fig. 3B20–B23). The inflammatory indices did not significantly correlate with the IL-2 *serum* concentrations (*P* > 0.05).

### Effects of Low-dose IL-2 in Psoriasis

#### Inflammatory Indicators After IL-2 Combination Therapies in Psoriasis

Next, we observed whether the Treg defects in psoriatic patients could be reversed with low-dose IL-2. The 45 psoriatic patients received methotrexate at 7.5 mg weekly for 12 weeks. From 13 to 24 weeks, they received methotrexate combined with three cycles of low-dose IL-2 therapy (0.5 MIU/d, subcutaneous injections). After treatment, inflammatory indicators of disease activity, including the leukocyte count, neutrophil count, ESR, CRP, and NLR in* serum*, decreased significantly compared with before treatment (*P* < 0.05). The lymphocyte count, platelet count, and PLR in* serum* did not change after treatment (*P* > 0.05; Table 5).

**Table 5:** Changes in the Inflammatory Indicator Levels Before and After Treatment [Mean ± SD, M(IQR)]

**Parameter**	**Before treatment**	**After treatment**	***t/Z***	***P***
Leukocytes (× 10^9^/L)	7.030 (3.410)	7.046 ± 1.589	−2.258	0.024
Neutrophil (× 10^9^/L)	4.920 (3.140)	4.714 ± 1.560	−2.664	0.008
Lymphocytes (× 10^9^/L)	1.947 ± 0.540	2.015 ± 0.559	0.555	0.581
Platelets (× 10^9^/L)	235 (189)	240 ± 58	−1.913	0.056
ESR (mm/h)	30 (63)	13 (16)	−5.696	<0.001
CRP (mg/L)	18.560 (43.645)	8.290 (16.855)	−4.809	<0.001
NLR	3.440 (1.670)	2.262 (1.166)	−4.656	<0.001
PLR	144.630 (96.175)	115.672 (38.526)	−1.834	0.067

*NLR* neutrophil-to-lymphocyte ratio, *PLR* platelet-to-lymphocyte ratio

#### Low-dose IL-2 Can Effectively Promote Treg Cells and Reverse the Th17/Treg Immune Imbalance in Psoriasis

**Fig. 4:**
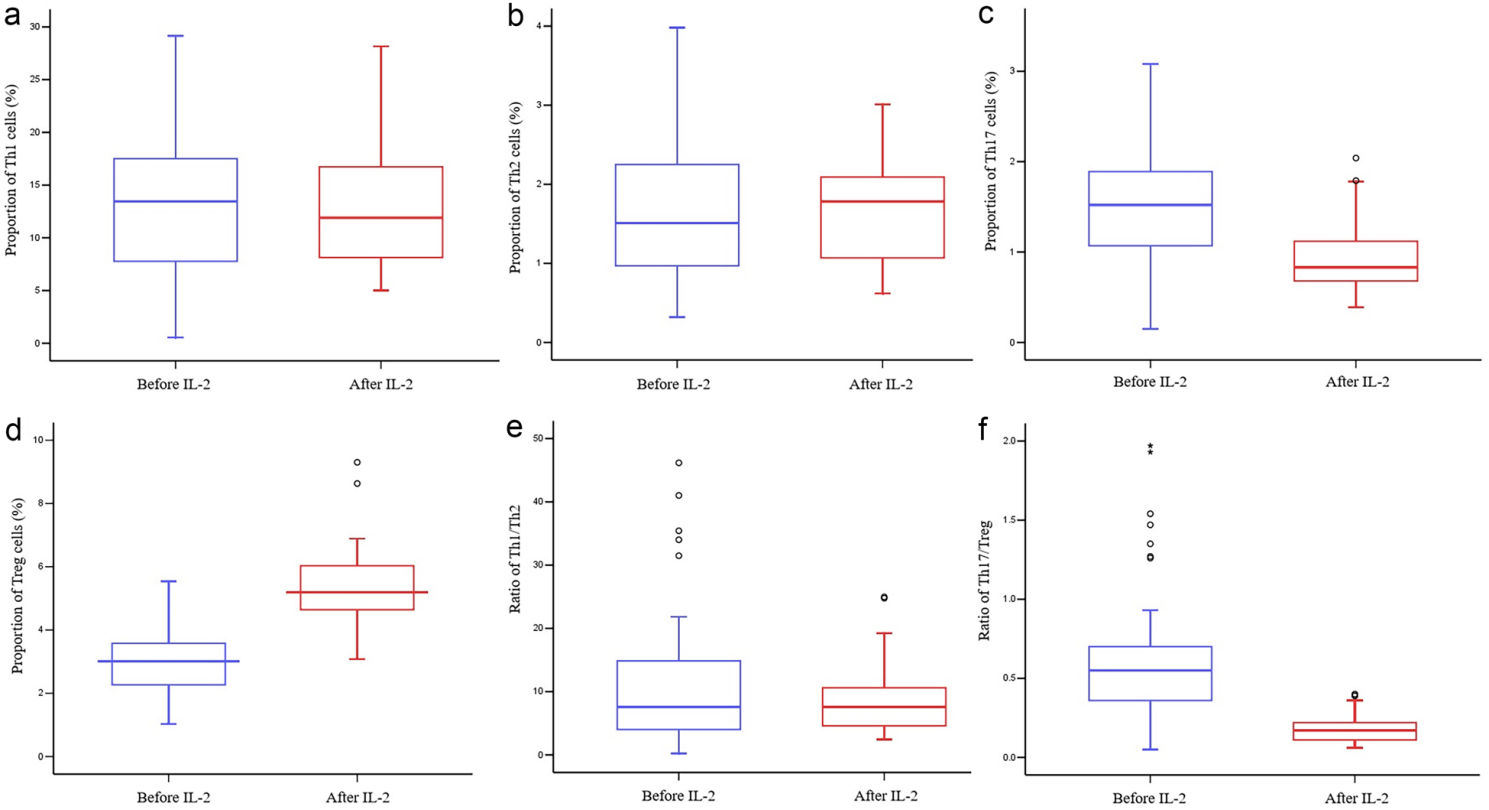
Proportions of **a** Th1 cells, **b** Th2 cells, **c** Th17 cells, and **d** Treg cells. Ratios of **e** Th1/Th2 and **f** Th17/Treg.

After IL-2 treatment, the percentage of Treg cells was greatly amplified (twofold increase) and decreased the Th17/Treg ratio (*P* < 0.001). The proportion of Th17 cells in peripheral blood was decreased after treatment (*P* < 0.001). The percentage of Treg cells was fivefold more than that of Th17 cells, which was the main reason for the decrease in the Th17/Treg ratio. No significant differences in Th1, Th2, or the Th1/Th2 ratio were observed after treatment (*P* > 0.05; Fig. 4).

#### Efficacy of Low-dose IL-2 Treatment on the Peripheral Cytokine Concentrations in Psoriasis

**Fig. 5:**
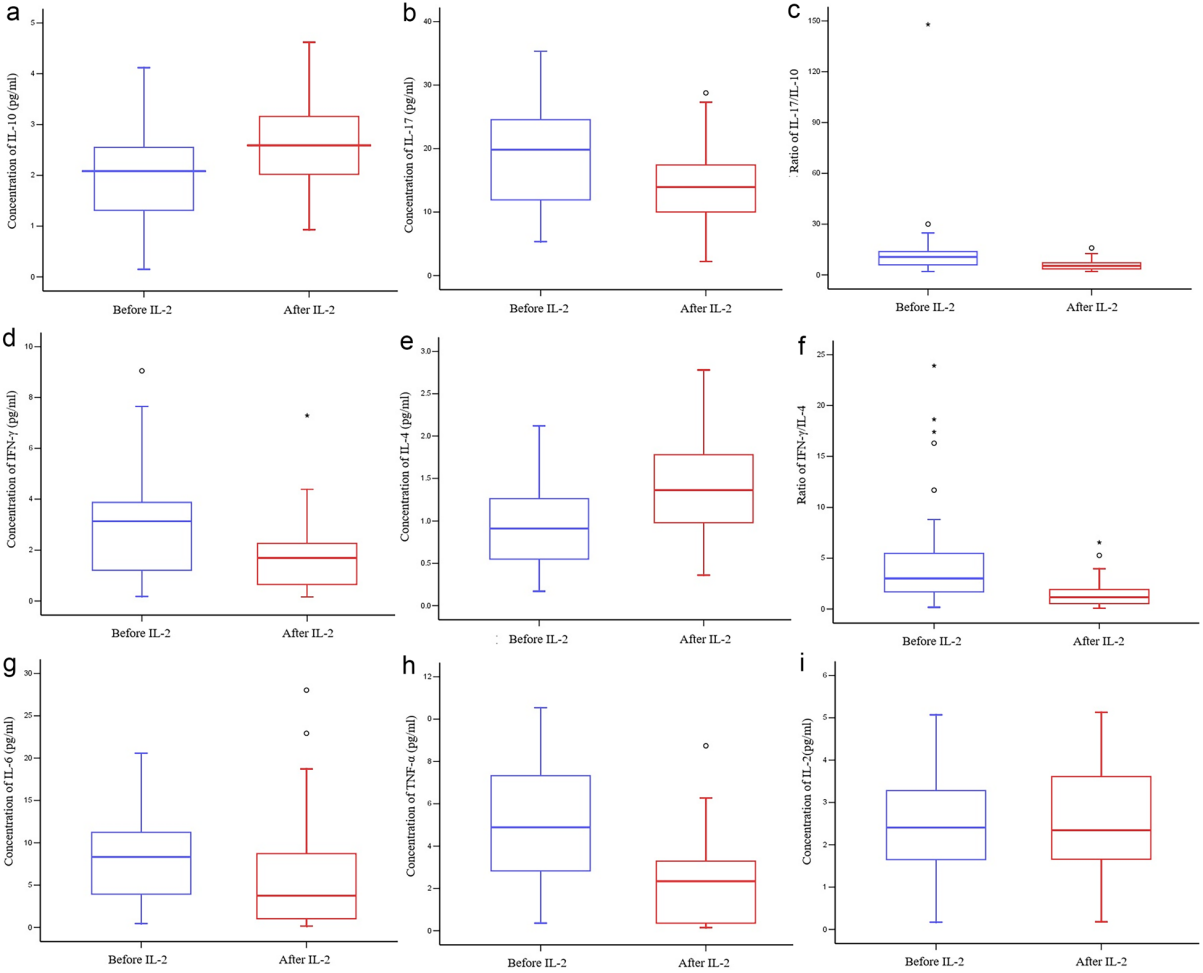
Concentrations of **a** IL-10, **b** IL-17, **d** IFN-γ, **e** IL-4, **G** IL-6, **h** TNF-α, and **i** IL-2. Ratios of **c** IL-17/IL-10 and **f** IFN-γ/IL-4.

Low-dose IL-2 treatment significantly increased the concentrations of anti-inflammatory cytokines (IL-10 and IL-4) among the psoriatic patients (*P* < 0.05). In contrast, the concentrations of pro-inflammatory factors (IL-6, IL-17, IFN-γ, and TNF-α) in peripheral blood were significantly decreased after treatment (*P* < 0.05). As a result, the IL-17/IL-10 and IFN-γ/IL-4 ratios were decreased after treatment (*P* < 0.001). However, no significant differences in the IL-2 concentration were observed between before and after the treatment (*P* > 0.05; Fig. 5).

### Clinical Efficacy and Safety of Low-dose IL-2 Therapy

After 24 weeks of treatment, the PASI 50 and PASI 75 were 68.89% and 72.22%, respectively. The ACR20 percentages were 35% after treatment (*P* < 0.05). Low-dose IL-2 was well tolerated, and most adverse events were mild and of a transient nature, suggesting a favourable safety profile. Two patients experienced injection-site reactions on day 4 of treatment. The reactions subsided spontaneously within 1 week. No significant differences in the alanine transaminase, blood urea nitrogen, or *serum* creatinine were observed between the psoriatic patients and the treatment groups.

## DISCUSSION

Th17/Treg immune imbalance has been reported to contribute to the pathogenesis of psoriasis [1–3]. Normally, naïve CD4^+^ T cells differentiate into Th17 and Treg cells. The Th17 cells produce IL-6, IL-17, and TNF-α to mediate the inflammatory reaction in psoriasis. Meanwhile, Treg cells produce suppressive cytokines such as IL-10, transforming growth factor-β, and cell–cell interaction, thereby playing an important role in immunosuppression. Th17 and Treg cells regulate each other to maintain immune balance [6–8].

In this study, the proportion of Th17 cells in peripheral blood was significantly increased in the patients with psoriasis compared with the healthy controls, and the Th17 cell proportion positively correlated with NLR, ESR, CRP, and PASI. Moreover, the Treg cell percentage was dramatically decreased and negatively correlated with NLR, ESR, CRP, and PASI. These changes resulted in a dramatic increase in the Th17/Treg ratio, which increased in the following order: PV < PA < EP < GPP. The Th17/Treg ratio positively correlated with NLR, ESR, CRP, and PASI. However, no significant differences in Th1, Th2, or the Th1/Th2 ratio were observed between the psoriasis and healthy groups. These results suggest that the percentages of Th17 and Treg cells are closely related to psoriasis disease severity.

Our findings agree with Ma *et al*. who reported Th17/Treg immune imbalance in PV patients [9]. However, Zhang *et al*. found that Th17 and Treg cells were increased in psoriatic patients in the peripheral circulation, both of which positively correlated with PASI score [10, 11]. These distinct results may stem from differences in disease states, Treg definition, the time frame from which the PASI scores were derived, and the investigated psoriasis subtypes [12, 13]. Overall, these studies suggest that Th17/Treg immune imbalance potentially contributes to psoriasis pathogenesis.

The immune imbalance between Th17 and Treg cells is associated with the disorder of pro-inflammatory and anti-inflammatory cytokines in psoriasis. In this study, the *serum* concentrations of IL-10 in the psoriasis group were decreased compared to the healthy group, and the IL-10 concentrations negatively correlated with NLR, ESR, CRP, and PASI. Reduction in IL-10 concentrations indirectly showed that Treg cells’ suppressive function was impaired, and this could not effectively resist the pro-inflammatory effect of increased Th17 cells secretion of IL-17. The *serum* concentrations of IL-17 were higher in the psoriatic patients compared to the healthy controls. As a result, the IL-17/IL-10 ratio was increased in the psoriasis group compared to the healthy group, and it positively correlated with NLR, PLR, ESR, CRP, and PASI. The increased IL-17/IL-10 ratio further confirms the imbalance of Th17/Treg cells in psoriasis.

IL-6 is a critical factor in the homeostatic balance between Th17 and Treg cells, and high concentrations of IL-6 increase Th17 cell production in cooperation with transforming growth factor-β and decrease Treg cell activity [14, 15]. In this study, the concentrations of IL-6, IFN-γ, and TNF-α were significantly elevated in the psoriasis group compared with the healthy group. The *serum* concentrations of IL-6 positively correlated with ESR and PASI; the *serum* concentrations of IFN-γ positively correlated with PASI, and the TNF-α *serum* concentrations positively correlated with NLR, ESR, CRP, and PASI. Yang *et al*. reported that the secretion of IFN-γ and TNF-α by Tregs was increased in psoriatic patients, suggesting that the conversion of Tregs to Th1 cells may impair Treg cell function, leading to the excessive activation of conventional T cells in autoimmune diseases [16]. Josefowicz *et al*. also reported the impaired function of Treg cells, leading to Th2 cell overactivation [17]. These Treg defects were associated with disease severity, and the recovery of Treg may be an effective method for the treatment of psoriasis.

Low-dose IL-2 therapy aims to compensate for the shortage of Treg cells and expand their population. Treg cells are more sensitive to IL-2 and require by far much lower doses of IL-2 for their stimulation because Treg cells expressing CD25 on the surface can form high-affinity IL-2 receptor complex, which is composed of α (CD25), β (CD122), and γ chain (CD132). In contrast, CD4^+^ conventional T cells, CD8^+^ T cells, or natural killer cells do not express CD25 and need high-dose IL-2 for stimulation [5, 18, 19]. IL-2 directly activates and phosphorylates the transcription factor STAT5, which upregulates the key transcription factor Forkhead helix protein 3 (Foxp3) of Treg cells, thereby inducing the differentiation of Treg cells and maintaining their activity [20, 21]. However, IL-2 activation of STAT5 can inhibit the expression of retinoid-related orphan receptor γt and then inhibit the differentiation of Th17 cells [22, 23]. As mentioned above, IL-2 is a crucial factor in the production and maintenance of Treg cells and reverses the imbalance of Th17/Treg cells.

Low-dose IL-2 has attracted attention in the treatment of autoimmune diseases. Clinical studies have shown that many immune-related diseases, such as systemic lupus erythematosus (SLE), rheumatoid arthritis, polymyositis/dermatomyositis, Sjögren’s syndrome, Treg cell defects, and Th17/Treg cell imbalance, may benefit from low-dose IL-2 treatment [24–28]. Humrich *et al*. used low-dose IL-2 to treat SLE with moderate-to-severe disease activity, supporting that Treg defects were associated with disease severity and could be corrected using low-dose IL-2, especially for active and refractory SLE [24]. More recently, in an open-label, multicentre phase I/IIa clinical trial that used low-dose IL-2 across 11 individual diseases suffering from autoimmune inflammatory conditions, the level of Treg cells in patients significantly increased (2.0 ± 0.6) fold, without effector T cell activation, and the clinical symptoms improved significantly [29]. 

In this study, 45 psoriatic patients were treated with low-dose IL-2 combined with methotrexate, which greatly amplified the percentage of Treg cells. The proportion of Th17 cells and the Th17/Treg ratio decreased significantly. No significant differences in Th1, Th2, or the Th1/Th2 ratio were observed after treatment. The percentage of Treg cells was fivefold more than that of Th17 cells, which means anti-inflammatory Tregs play important roles in restoring the Th17 and Treg cell balance in psoriasis. Wang *et al*. reported that in PA patients that exhibited low Treg numbers, low-dose IL-2 combination with conventional therapy not only increased the absolute numbers of Tregs but also rapidly alleviated PA disease activity as indicated by the disease activity score without any apparent side effects [30]. Therefore, these findings strengthen the concept that low-dose IL-2 corrects Treg cell defects, promotes Treg cell expansion, and restores immune balance.

At the same time, low-dose IL-2 treatment also significantly increased the concentrations of anti-inflammatory cytokines (IL-4 and IL-10) but decreased the IFN-γ/IL-4 and IL-17/IL-10 ratios and regulated the production of pro-inflammatory factors (IFN-γ, IL-6, IL-17, and TNF-α), leading to significant decreases in the IFN-γ/IL-4 and IL-17/IL-10 ratios. These results indicate that low-dose IL-2 can not only restore the activity of Treg cells but also improve the pro-inflammatory environment in psoriasis and rebuild the immune balance.

Low-dose IL-2 in psoriatic patients can reduce disease activity by promoting the expansion of circulating Tregs and restoring immune balance. After low-dose IL-2 treatment, the clinical manifestations of psoriasis improved, and the PASI 50 and PASI 75 were 68.89% and 22.22% at week 24, respectively. The ACR20 percentages were 35% after treatment in PA. Low-dose IL-2 was well tolerated, and most adverse events were mild and of a transient nature, suggesting a favourable safety profile. He *et al*. reported low-dose IL-2 therapy in a randomised, double-blind, placebo-controlled clinical trial in 60 patients with active SLE. At week 12, the SLE responder index-4 response rates were 55.17% and 30.00% for the IL-2 and placebo groups, respectively. The prednisone dose was reduced by more than 50% at week 24. The levels of anti-dsDNA antibodies were decreased in the IL-2 group compared with the placebo group, whereas the complement C3 and C4 levels were increased. An expansion of the Treg cells was observed in this study. The number of Treg in peripheral blood was increased, the Tfh and Th17 cells were decreased, and the (Tfh+Th17)/Treg ratio was decreased [31, 32].

After administering low-dose IL-2 for psoriasis, it also decreased the inflammatory indicators of disease activity, including the leukocyte count, neutrophil count, ESR, CRP, and NLR in *serum* after treatment. NLR is a new inflammatory index calculated by dividing the number of neutrophils by the number of lymphocytes. Neutrophils reflect systemic inflammation, and lymphocytes reflect the immune status in psoriatic patients [33, 34]. We found that circulating NLR was significantly higher in the psoriasis group compared to the healthy group. NLR positively correlated with the levels of Th17, IL-17, and TNF-α along with the Th17/Treg and IL-17/IL-10 ratios. NLR negatively correlated with Treg cells and IL-10 concentrations. After low-dose IL-2 treatment, NLR levels decreased in the *serum* of psoriatic patients. Kim *et al*. reported that NLR was increased in psoriatic patients compared to the control group and that NLR positively correlated with PASI [35]. Altogether, these results indicate that NLR combined with conventional inflammatory indicators is useful markers for evaluating systemic inflammation in psoriatic patients. Thus, NLR may serve as simple, convenient, and cost-effective biomarkers to monitor the course of psoriasis before and after systemic therapy [33].

In conclusion, inflammatory diseases are associated with insufficient Tregs in psoriasis, leading to Th17/Treg immune imbalance, which contributes to psoriasis pathogenesis, supporting the regulation of Th17/Treg imbalance as a potential psoriasis treatment. Notably, low-dose IL-2 not only selectively activated and expanded Treg cells but also, with conventional therapies, may improve the anti-inflammatory environment and rebuild immune balance in psoriatic patients. Low-dose IL-2 can rapidly alleviate disease activity and improve clinical symptoms significantly. Low-dose IL-2 showed good safety and was well tolerated. As a new immunomodulatory therapy, low-dose IL-2 may be an effective treatment option for psoriatic patients.

### Supplementary Information

Below is the link to the electronic supplementary material.

Supplementary file1 (DOC 3990 KB)

## Data Availability

All data relevant to the study were included in the article and are available upon reasonable request.
