# Supplementary material for: Low-dose Interleukin-2 For Psoriasis Therapy Based on the Regulation of Th17/Treg Cell Balance in Peripheral Blood
Source: Inflammation. 2023 Aug 18;46(6):2359–73. doi: 10.1007/s10753-023-01883-6 (PMC10673739; doi:10.1007/s10753-023-01883-6)
Supplement: Supplementary file 1 — Supplementary file1 (DOC 3990 KB) [file 10753_2023_1883_MOESM1_ESM.doc]

**Tables and Figure legends**

**Table 1 Baseline characteristics and inflammatory parameters of all subjects [mean±SD,M(IQR)]**


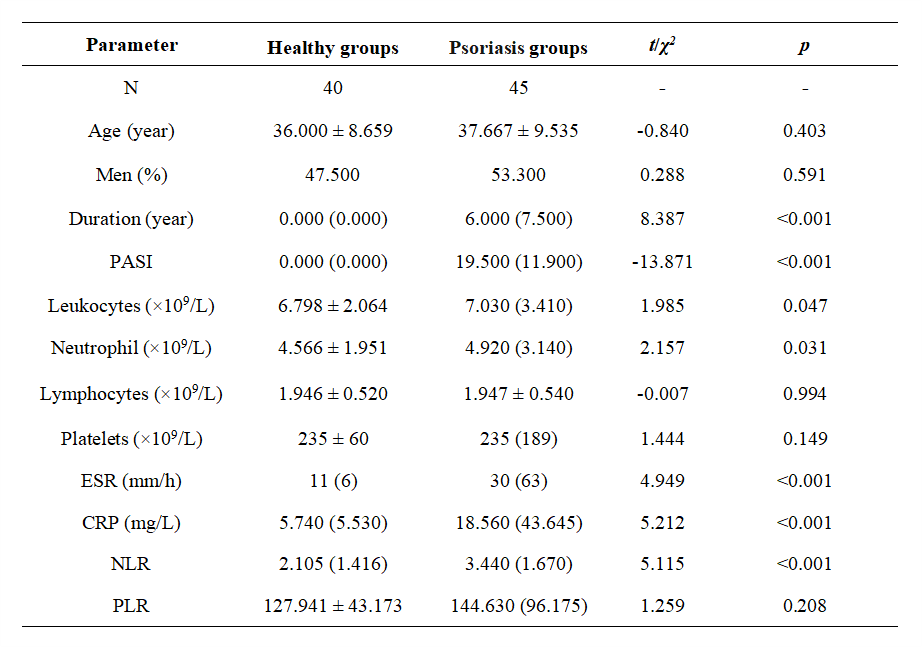


NLR, neutrophil-to-lymphocyte ratio; PASI, psoriasis area and severity index; PLR, platelet-to-lymphocyte ratio

**Table 2 Clinical characteristics and inflammatory parameters among the different psoriasis groups [mean±SD,M(IQR)]**


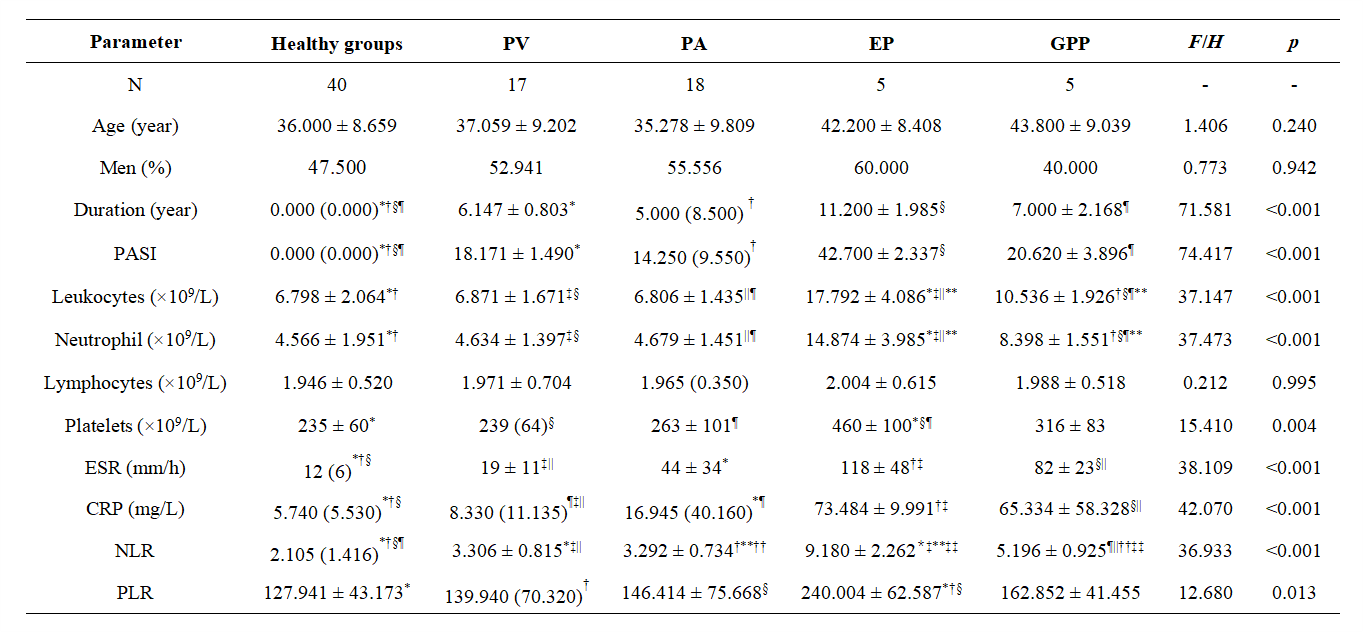


Values are shown as mean ± standard deviation or median (interquartile range). (^*†‡§||¶**††‡‡^*P* < 0.05). NLR, neutrophil-to-lymphocyte ratio; PASI, psoriasis area and severity index; PLR, platelet-to-lymphocyte ratio

**Table 3 Differences in CD4^+^ T cell subset numbers in the psoriasis and healthy group[mean±SD,M(IQR)]**


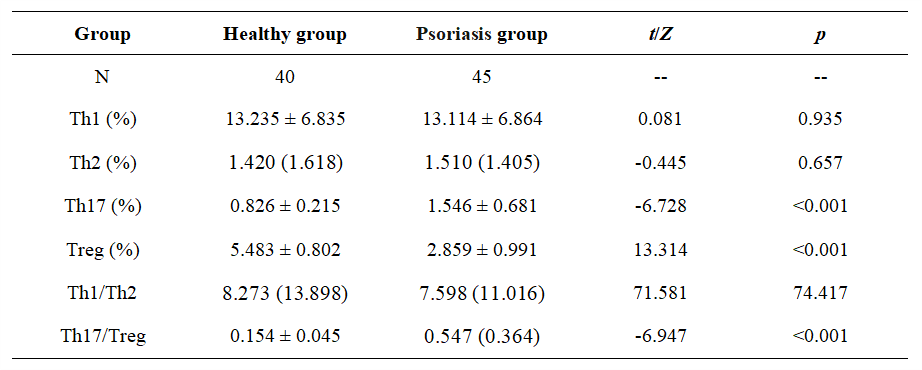


**Table 4 Differences in cytokine levels between the psoriasis and healthy groups (pg/ml) [mean±SD,M(IQR)]**


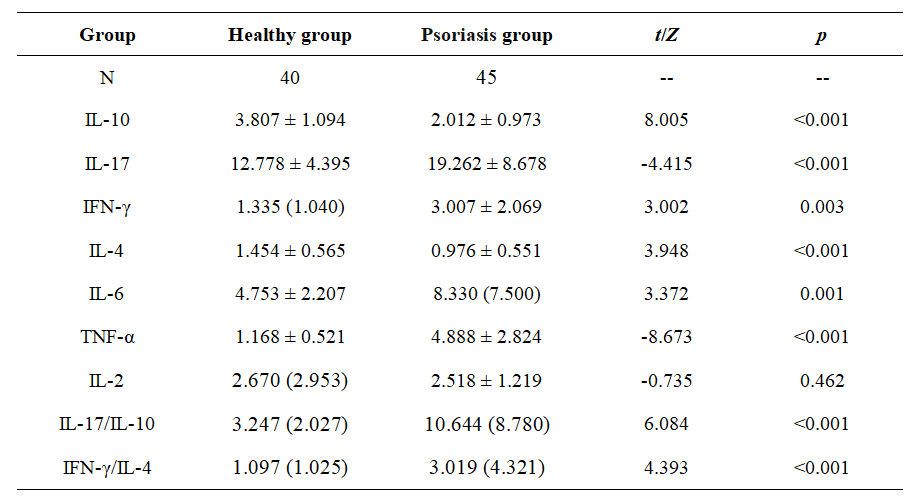


**Table 5 Changes in the inflammatory indicator levels before and after treatment [mean±SD, M(IQR)]**

**
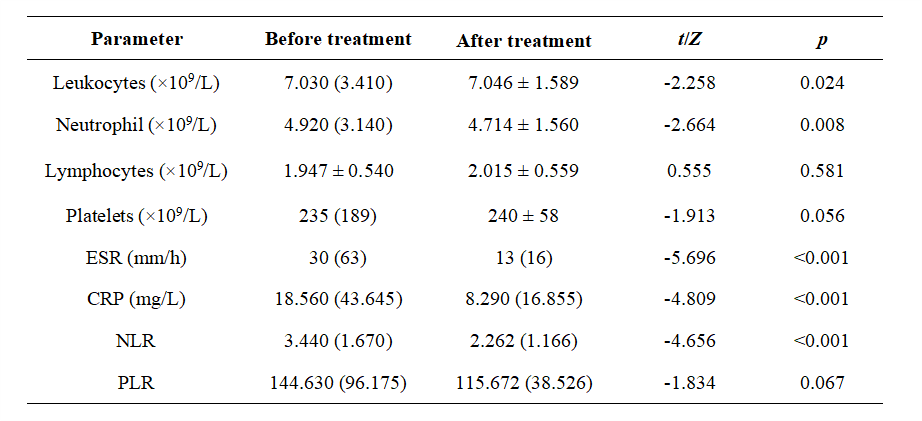
**

NLR, neutrophil-to-lymphocyte ratio; PLR, platelet-to-lymphocyte ratio


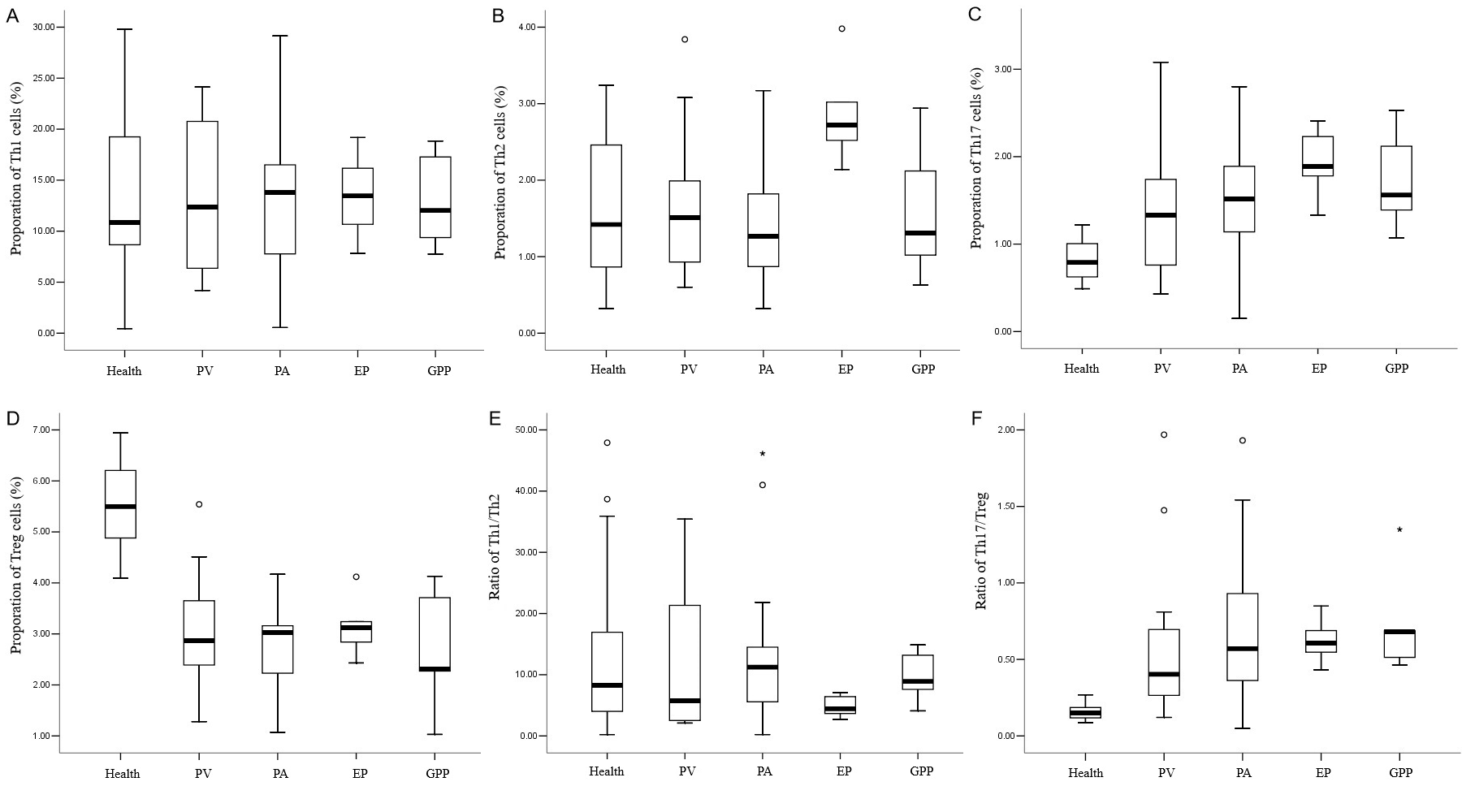


**Fig. 1** Comparison of CD4^+^ T lymphocyte subsets in peripheral blood between the psoriasis and control groups. (A–D) Percentages of Th1, Th2, Th17, and Treg cells in the peripheral blood of the PV, PA, EP, and GPP patients compared with the healthy controls. (E, F) Th1/Th2 and Th17/Treg ratios in the four types of psoriatic patients compared with the controls.


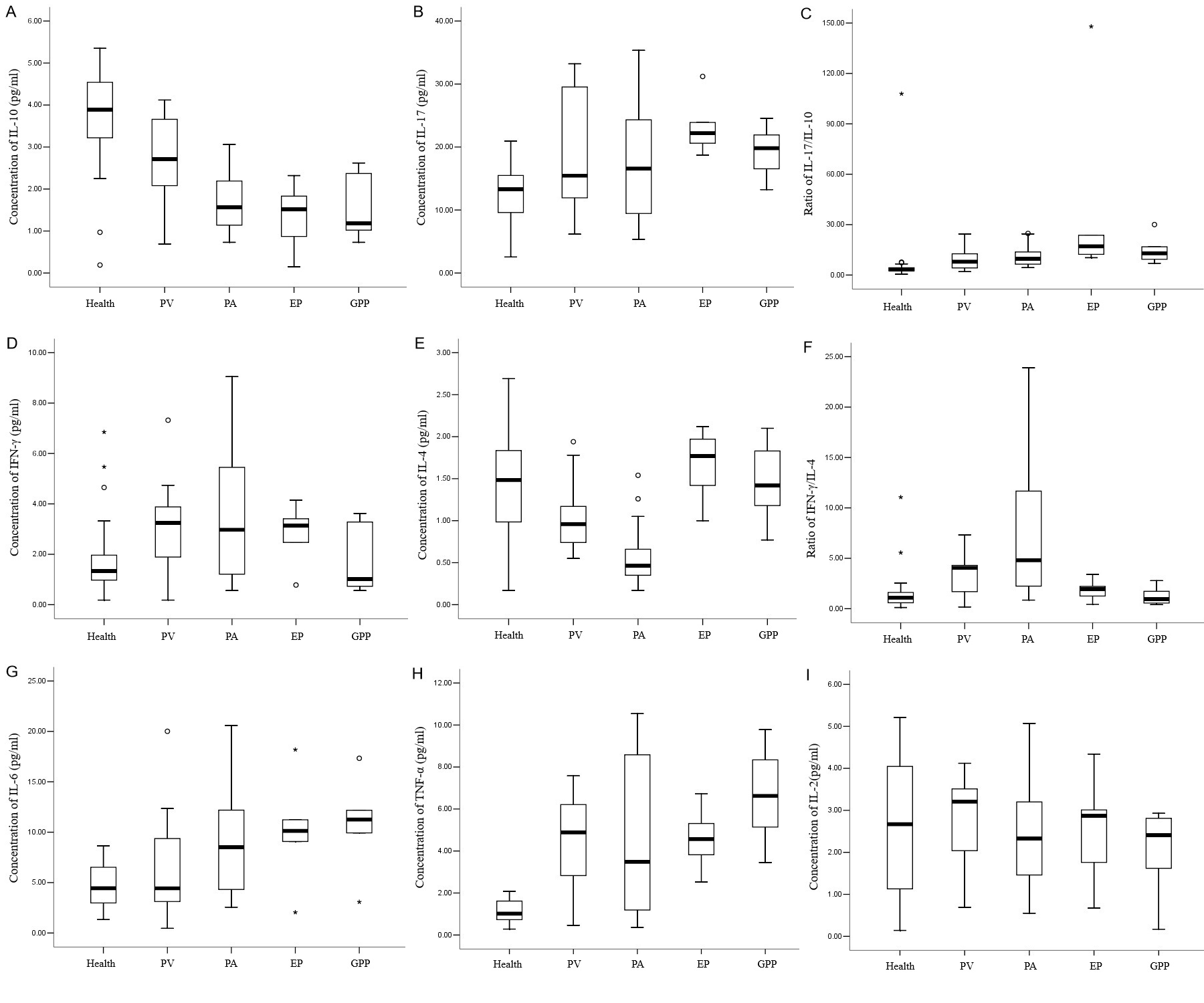


**Fig. 2** Characteristics of cytokines in psoriatic patients. (A, B, D, E, G, H) The serum concentrations of IL-10, IL-17, IFN-γ, IL-4, IL-6, and TNF-α in the PV, PA, EP, and GPP patients compared to the healthy controls. (C, F) The ratios of IL-17/IL-10 and IFN-γ/IL-4 in four types of psoriatic patients compared with the controls.


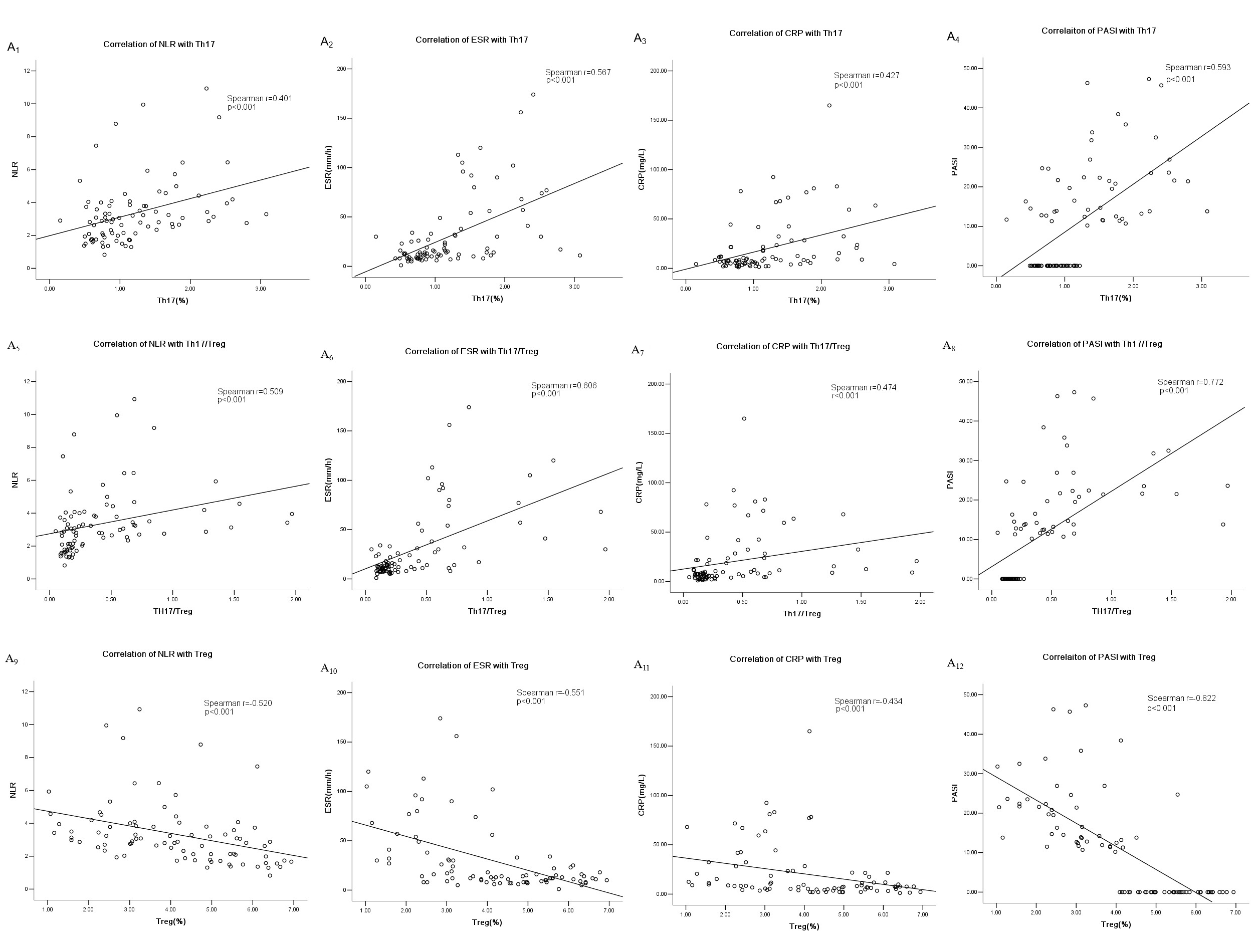


**Fig. 3A** Correlation analysis of Th17, Treg, and the Th17/Treg ratio with NLR, ESR, CRP, and PASI in the psoriasis group. NLR, neutrophil-to-lymphocyte ratio; PASI, psoriasis area and severity index

*
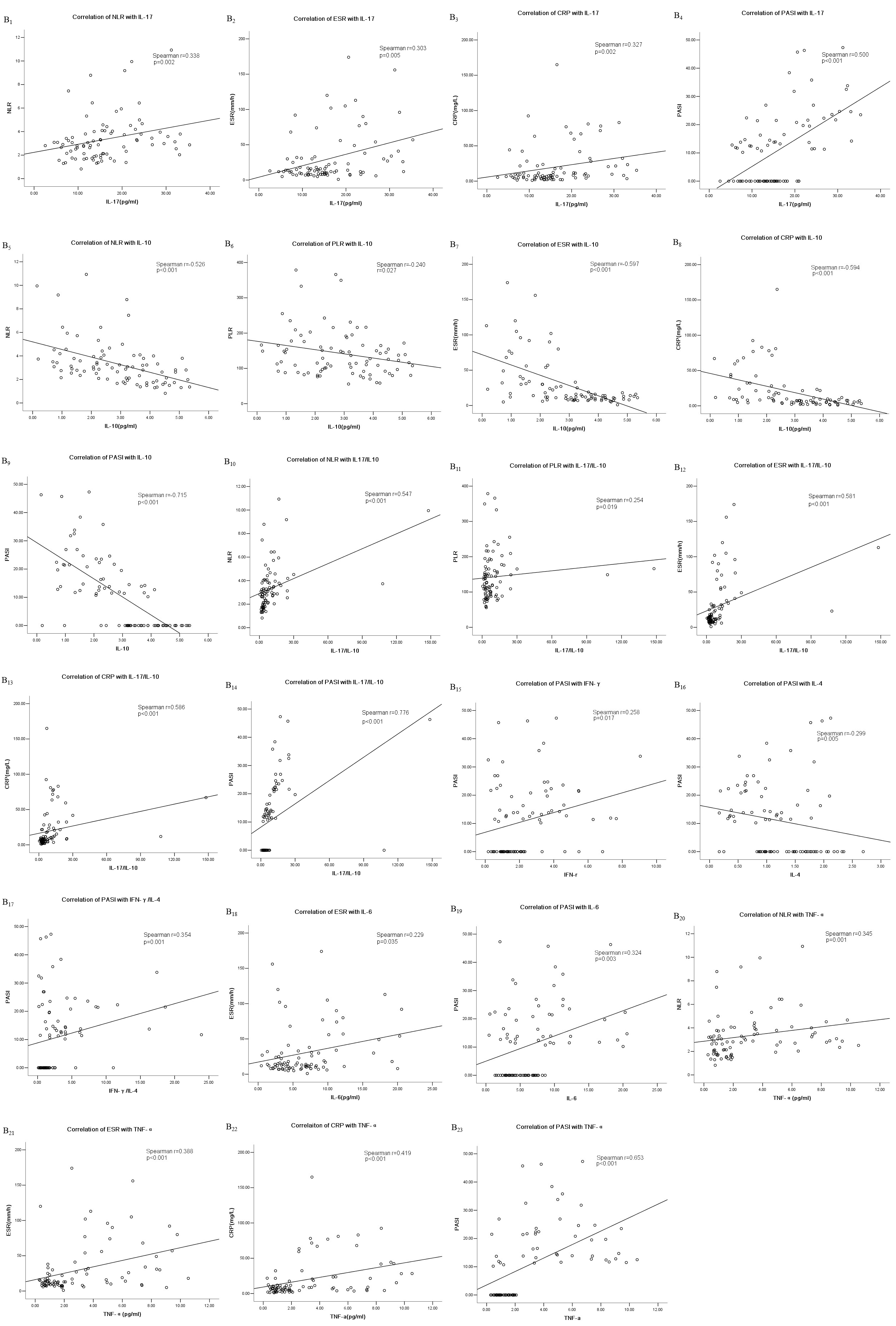
*

**Fig. 3B** Correlation analysis of IL-17, IL-10, IL-4, IL-6, IFN-γ, TNF-α, the IFN-γ/IL-4 ratio, and the IL-17/IL-10 ratio with NLR, PLR, ESR, CRP, and PASI in the psoriasis group. NLR, neutrophil-to-lymphocyte ratio; PASI, psoriasis area and severity index; PLR, platelet-to-lymphocyte ratio


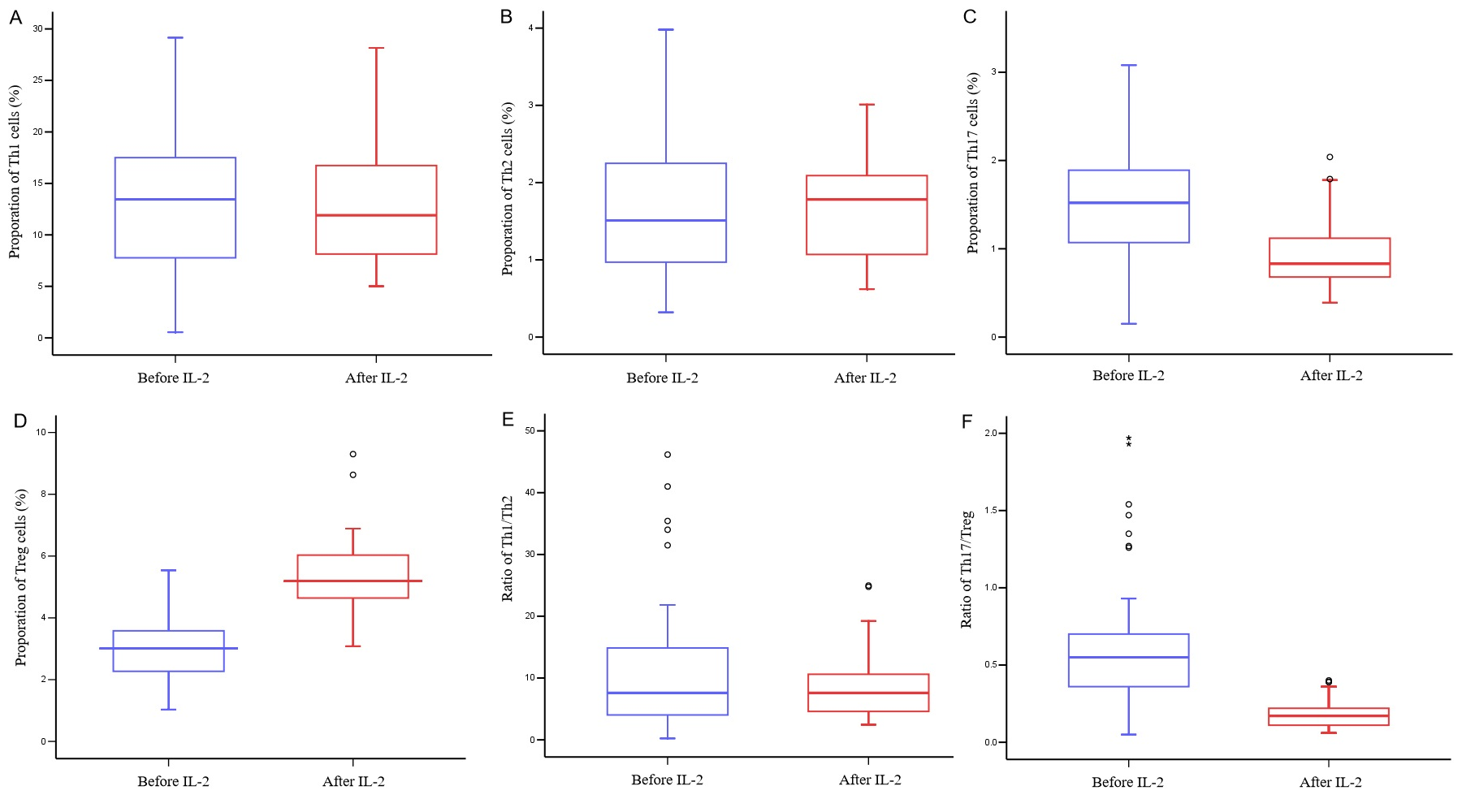


**Fig. 4** The changes of CD4^+^ T lymphocyte subsets in peripheral blood before IL-2 treatment (week 0) and after IL-2 combination therapies (week 24) for psoriasis. After IL-2 treatment, the percentage of Treg cells was amplified, and the ratio of Th17/Treg cells was restored.

***
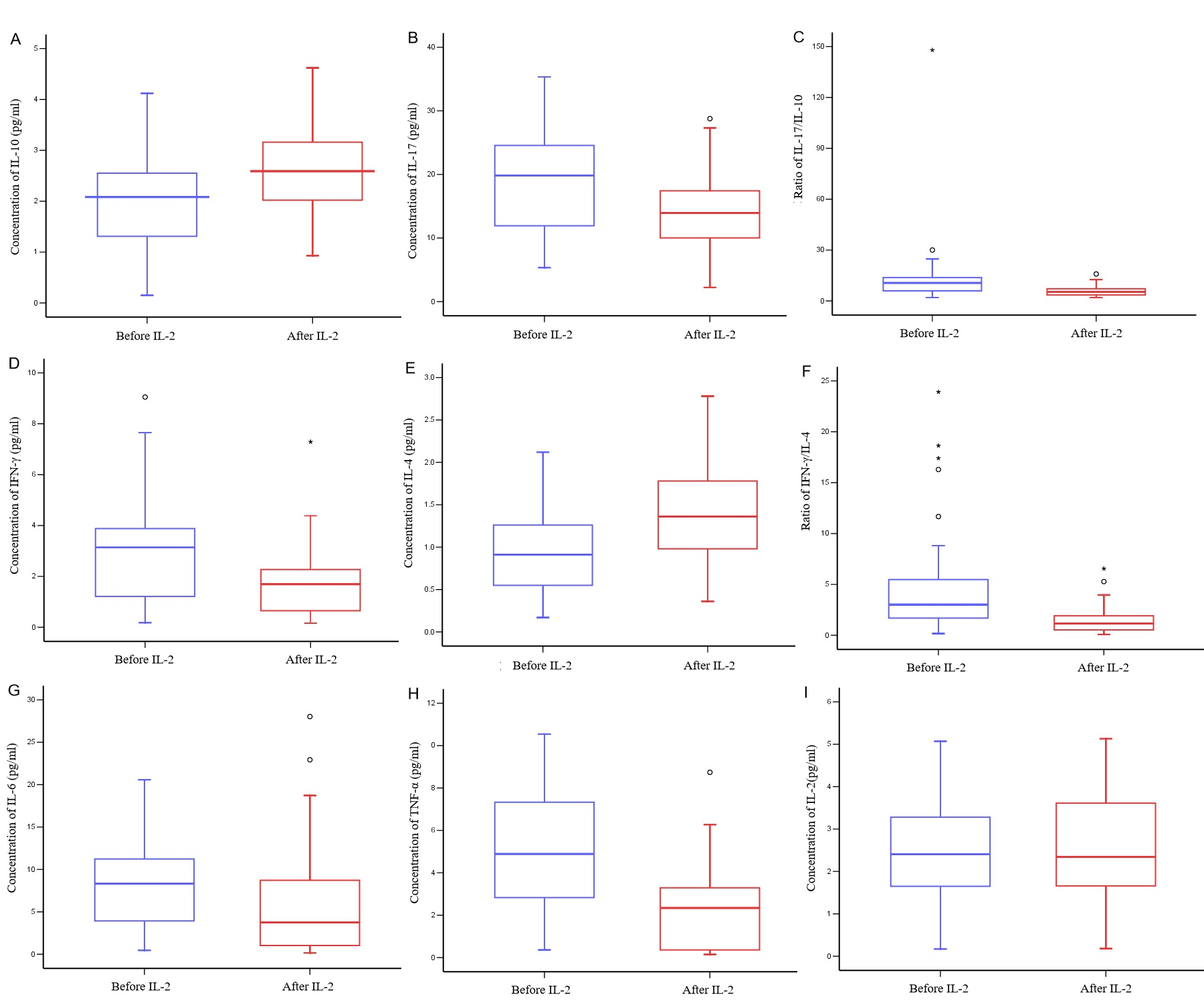
***

**Fig. 5** The changes of cytokine concentrations in peripheral blood before IL-2 treatment (week 0) and after IL-2 combination therapies (week 24). After IL-2 treatment, anti-inflammatory cytokines (IL-10 and IL-4) were increased, and the IL-17/IL-10 and IFN-γ/IL-4 ratios were decreased.
